# Supplementary material for: Proteogenomic Analysis Provides Novel Insight into Genome Annotation and Nitrogen Metabolism in Nostoc sp. PCC 7120
Source: Microbiol Spectr. 2021 Sep 15;9(2):e00490-21. doi: 10.1128/Spectrum.00490-21 (PMC8557916; doi:10.1128/Spectrum.00490-21)
Supplement: SUPPLEMENTAL FILE 10 — Supplemental material. Download SPECTRUM00490-21_Supp_10_seq9.pdf, PDF file, 2.4 MB [file spectrum00490-21_supp_10_seq9.pdf]

Supplemental Figures

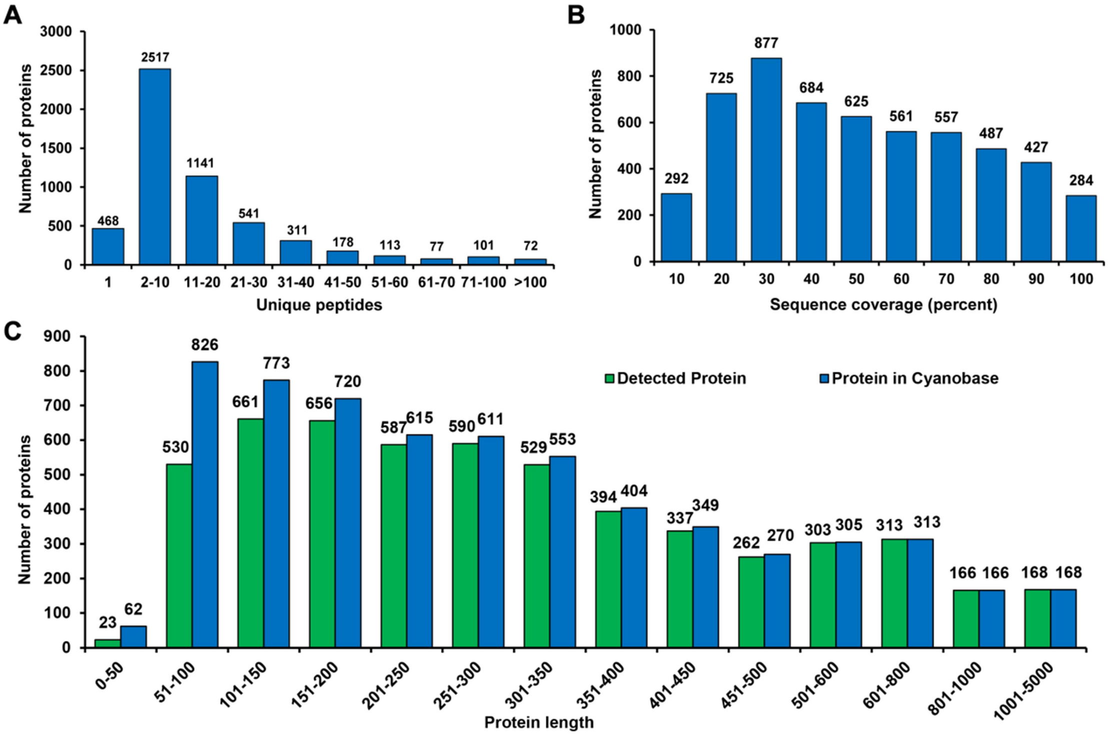

Figure S1. Overview of proteomic data.

A. Distribution of the number of unique peptides for all identified proteins. **B.** Distribution of the sequence coverage for all identified proteins. **C.** Protein length distribution of identified proteins.

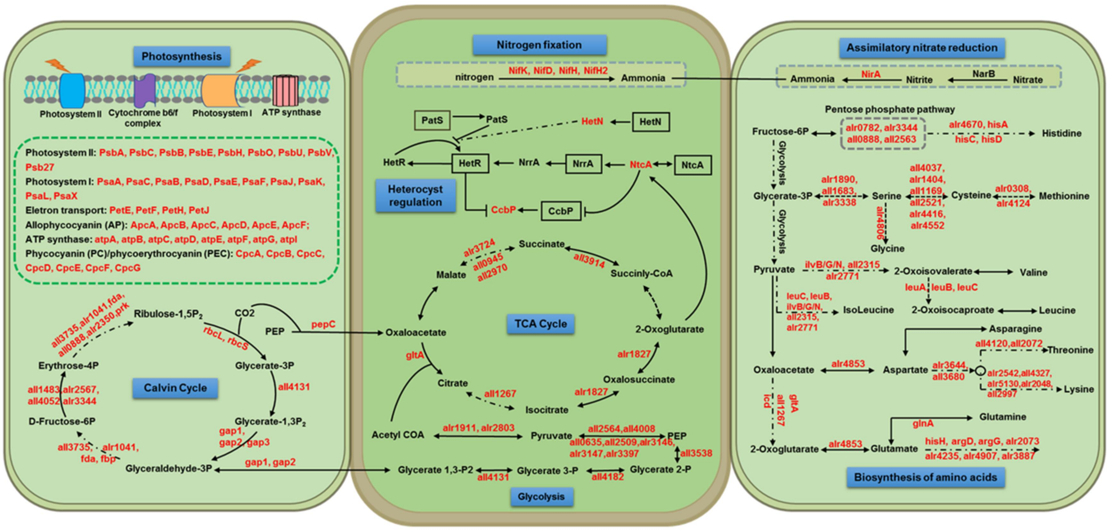

Figure S2. Overview of the modified proteins involved in diverse carbon and nitrogen metabolic pathways in *Nostoc* 7120.

The identified modified proteins are highlighted in red.

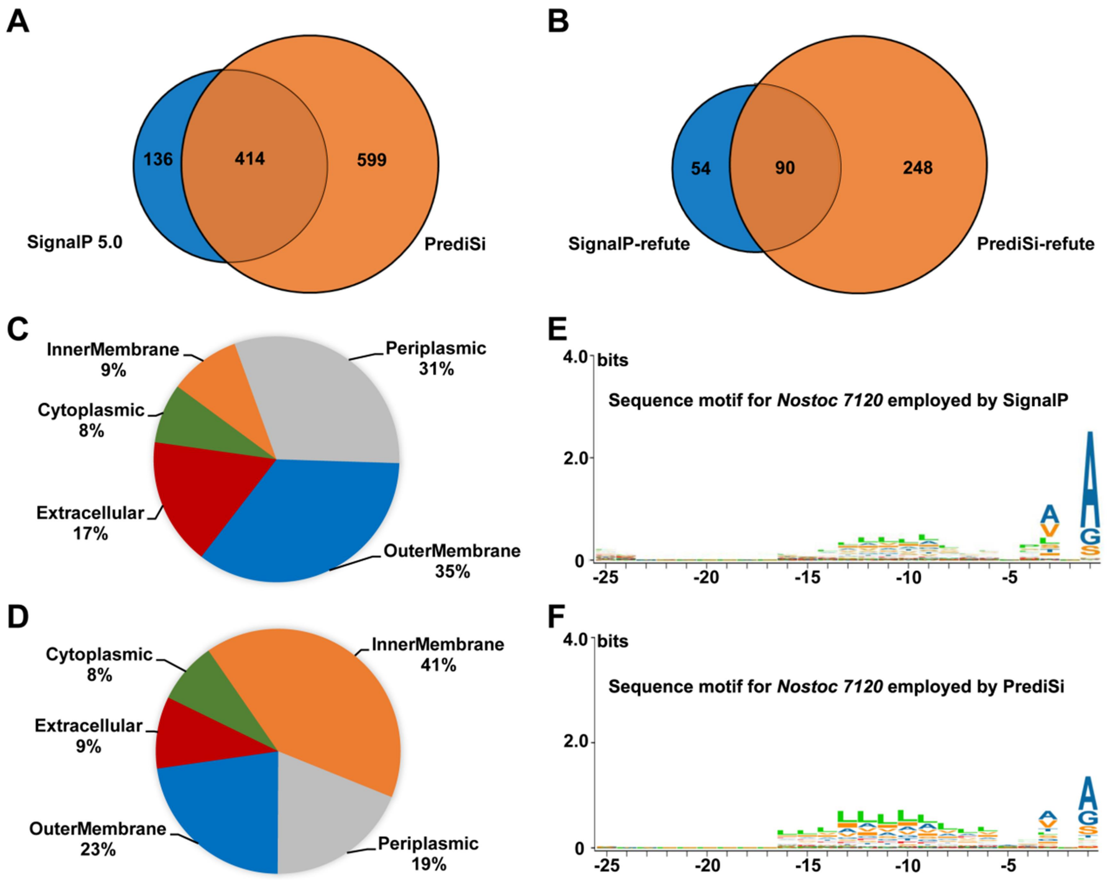

Figure S3. Signal peptide prediction in *Nostoc* 7120.

A. Venn diagram showing the number of signal peptides predictions by PerdiSi and SignalP. **B.** Number of signal peptide predictions by SignalP (25) and PrediSi (110) refuted according to the signal cleavage site of the identified peptides upstream. **C.** Subcellular localization of identified proteins containing signal peptides predicted by SignalP 5.0. **D.** Subcellular localization of identified proteins containing signal peptides predicted by PrediSi. **E.** Motif of signal peptides predicted by SignalP 5.0. **D.** Motif of signal peptides predicted by PrediSi.

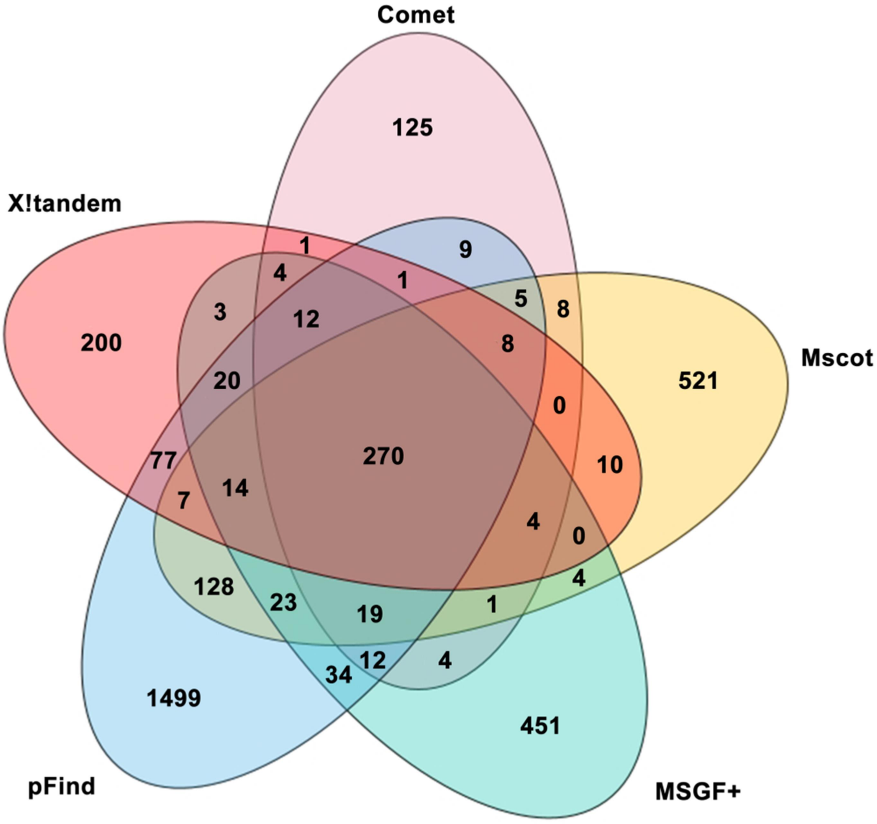

Figure S4. Venn diagram of novel peptides identified by different search engines.

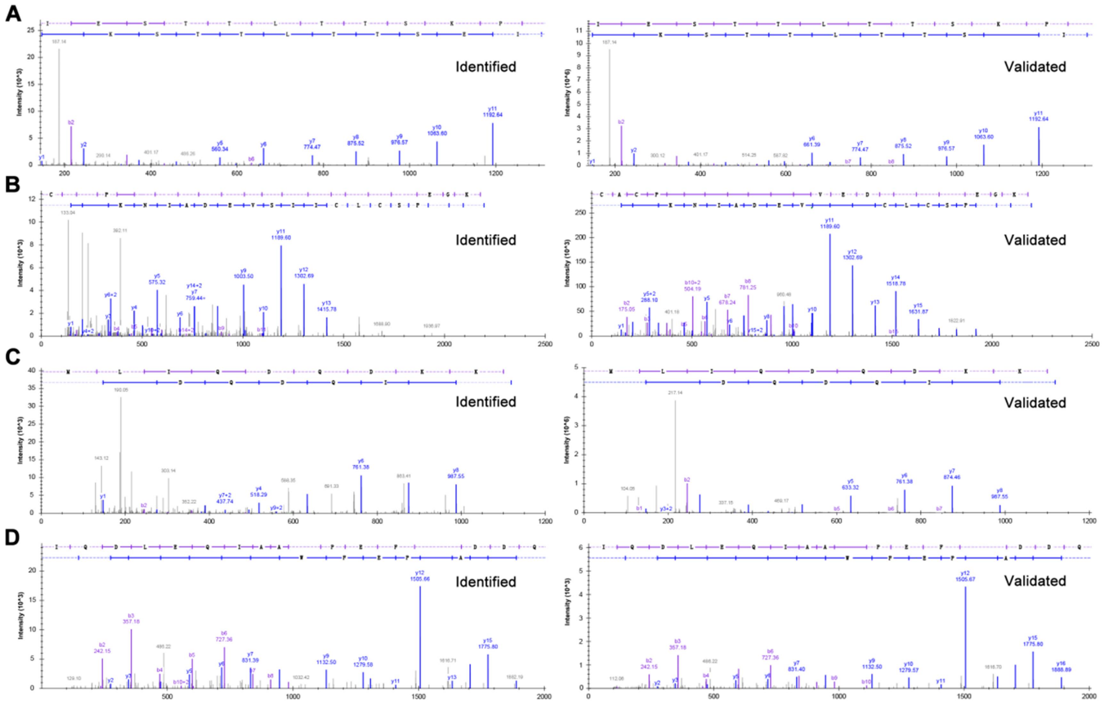

Figure S5. Validation of novel peptides by comparing the MS spectra of the peptides identified from proteogenomic analysis (Identified) to those of synthetic peptides (Validated).

A. MS spectra of peptides from a novel gene located in the intergenic region. **B.** MS spectra of peptides from a novel gene located on the opposite strand. **C.** MS spectra of peptides from a revised gene of alr5269 with N-terminal extension. **D.** MS spectra of peptide from a revised gene of all0273 with N-terminal extension with a nontraditional initiation codon (ATT).

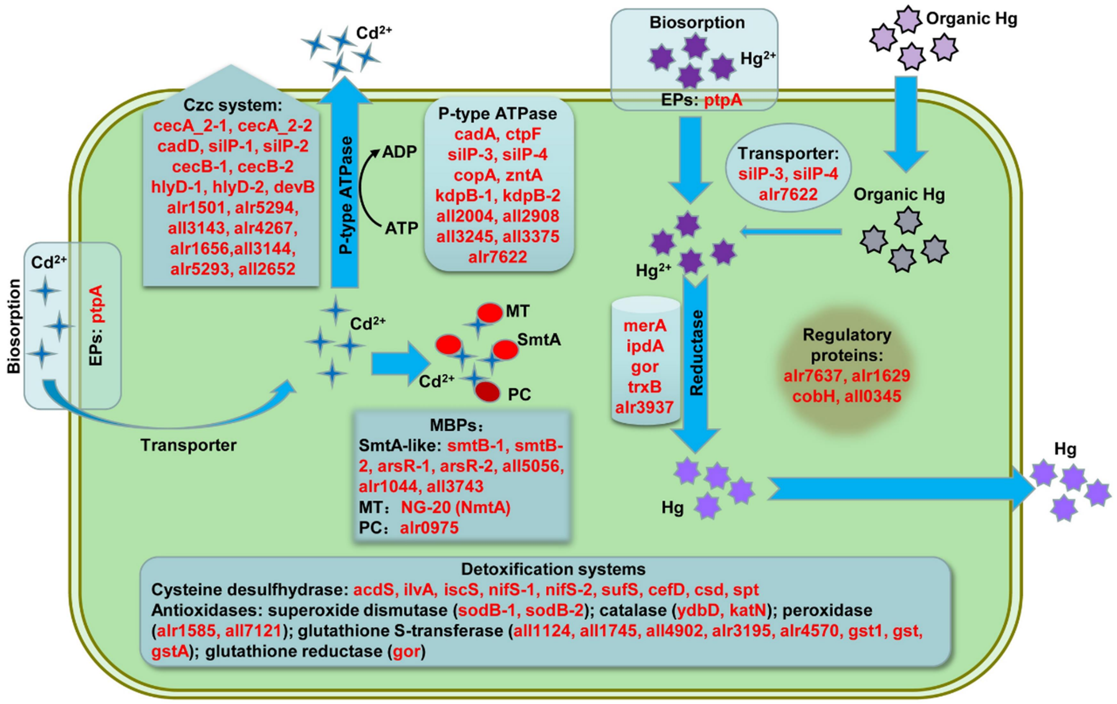

Figure S6. Schematic diagram of Cd- and Hg-related proteins in *Nostoc* 7120

The Cd- and Hg-related proteins identified in our proteogenomic analysis are highlighted in red.
